# Leaf beetle diversity on a Southeast Asian continental island: Taxonomy, DNA barcoding, and preliminary evolutionary insights from Cat Ba Island, Vietnam

**DOI:** 10.1371/journal.pone.0351706

**Published:** 2026-06-18

**Authors:** Dinh Thi Nguyen, Loan Thi Ho

**Affiliations:** Department of Nature Conservation, Institute of Biology, Vietnam Academy of Science and Technology, Hanoi, Vietnam; Laboratoire de Biologie du Développement de Villefranche-sur-Mer, FRANCE

## Abstract

Continental shelf islands in Southeast Asia remain poorly studied with respect to insect diversity and evolutionary history. Here, we provide an integrative assessment of species diversity, DNA barcode variation, diversification dynamics, and historical biogeography of leaf beetles (Coleoptera: Chrysomelidae) on Cat Ba Island, northern Vietnam. Based on field surveys, 36 morphospecies operational taxonomic units (OTUs) belonging to 30 genera and five subfamilies were documented, with Galerucinae representing the most species-rich lineage. DNA barcoding of the mitochondrial COI gene generated 31 Barcode Index Numbers (BINs), most of which represent new records in the Barcode of Life Data System, highlighting substantial undocumented genetic diversity. COI-based analyses were further used to explore broad temporal and evolutionary patterns. Within this exploratory framework, divergence-time estimation under a relaxed molecular clock suggests that major lineages may have originated during the early–middle Miocene, predating the formation of the present-day island. Diversification analyses support relatively constant rates through time with low inferred extinction, consistent with expectations for continental shelf island systems shaped by repeated connectivity and isolation. Model-based biogeographic analyses indicate predominantly localized ancestral ranges, with Cat Ba Island and adjacent mainland regions playing recurrent roles in the assembly of the fauna. Together, these results provide baseline taxonomic and genetic data for a poorly known insular insect assemblage while offering a preliminary evolutionary context that should be interpreted with caution and that can serve as a foundation for future biodiversity monitoring and comparative studies in dynamic island–mainland systems.

## Introduction

Islands have long been recognised as natural laboratories for investigating the processes that generate and maintain biodiversity, including colonisation, isolation, diversification and extinction [1,2]. Classical island biogeography theory emphasised the roles of island area and isolation in shaping species richness [3], whereas more recent frameworks incorporate geological history, temporal dynamics and evolutionary processes to explain patterns of insular diversity [4,5]. From a conservation perspective, islands are of particular importance because they often harbour unique species assemblages while being especially vulnerable to habitat disturbance and environmental change.

Continental shelf islands differ fundamentally from oceanic islands in both origin and evolutionary dynamics [6]. Rather than forming through volcanic processes, continental islands represent fragments of former mainland landscapes that have undergone episodic isolation driven primarily by sea‐level fluctuations [7]. As a consequence, their biotas are often assembled through repeated cycles of dispersal, persistence and limited in situ diversification, producing composite faunas with heterogeneous evolutionary histories [8,9]. Despite their relevance for understanding biodiversity assembly and persistence, the island systems have received comparatively little attention in insect conservation studies, particularly in Southeast Asia.

Southeast Asia is characterised by a complex geological and climatic history, marked by tectonic reorganisation and pronounced Quaternary sea‐level oscillations that repeatedly connected and isolated landmasses across the Sunda Shelf [7,10]. These processes are expected to have had profound effects on the distribution and diversity of terrestrial organisms. However, integrative studies that combine biodiversity surveys with molecular and evolutionary approaches remain scarce for insects, even though insects constitute the majority of terrestrial biodiversity and are increasingly recognised as being at risk from habitat loss and environmental change.

Leaf beetles (Coleoptera: Chrysomelidae) provide an excellent model for addressing these knowledge gaps. As one of the most speciose beetle families, Chrysomelidae comprise more than 35,000 described species worldwide [11–14]. Most species are phytophagous and often exhibit host‐plant specialisation, traits that can restrict dispersal and promote population differentiation under geographic isolation [15,16]. These characteristics make leaf beetles particularly suitable for assessing how island systems contribute to regional insect diversity and for generating baseline data relevant to conservation planning.

Cat Ba Island, located in the Gulf of Tonkin in northern Vietnam, forms part of the Ha Long–Cat Ba limestone archipelago and represents a continental shelf island system. Geological evidence indicates that this region has experienced repeated phases of subaerial exposure and marine inundation since the late Neogene, driven by glacial–interglacial sea‐level fluctuations [9,17]. Cat Ba Island is recognised for its high conservation value and is protected as both a national park and a UNESCO Biosphere Reserve. Nevertheless, the diversity and evolutionary composition of its insect fauna remain poorly understood, and no previous study has integrated species diversity data with molecular and evolutionary analyses for leaf beetles on the island.

In this study, we provide the first integrative assessment of leaf beetle diversity on Cat Ba Island by combining field surveys, morphological taxonomy and DNA barcoding with divergence‐time estimation and historical biogeography. Specifically, we aim to: (1) document patterns of species diversity and community composition; (2) generate DNA barcode reference data to support species identification and future biodiversity monitoring; and (3) place the island fauna in a broader evolutionary and spatial context. By explicitly linking biodiversity patterns with evolutionary history, this study provides essential baseline information for insect conservation on continental shelf islands in Southeast Asia.

Rather than aiming to resolve deep phylogenetic relationships or to test explicit macroevolutionary hypotheses, this study is designed to provide a robust baseline of taxonomic, genetic and evolutionary information for a poorly documented insular insect fauna. The analytical framework adopted here prioritises methodological transparency and data generation, with evolutionary inferences interpreted cautiously and at a coarse-grained level, consistent with the objectives of broad-scope biodiversity and data-driven studies. To our knowledge, this represents the first integrative study of insect diversity on a continental shelf island in Vietnam, providing a novel baseline for linking biodiversity patterns with evolutionary history in this region.

## Materials and Methods

### Study area and sampling

Cat Ba National Park is situated within the mainland–island complex of northern Vietnam and constitutes the core area of the Ha Long–Cat Ba Biosphere Reserve. The park is characterised by extensive limestone karst formations, evergreen tropical forests, and a high degree of habitat heterogeneity. Owing to these features, it harbours exceptionally rich biodiversity, including numerous endemic and threatened taxa, and plays a crucial role in regional conservation initiatives [18,19].

**Fig 1:**
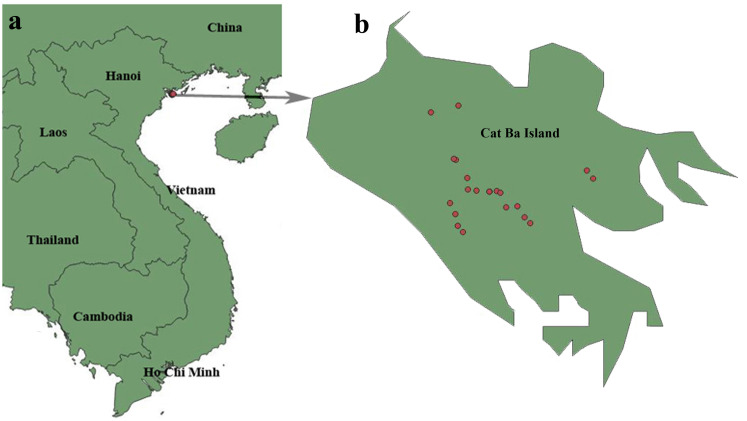
Sampling locations of Chrysomelidae on Cat Ba Island. (a) Regional map showing the location of Cat Ba Island within northern Vietnam (scale 1:9371119).(b) Detailed map of Cat Ba Island indicating sampling sites (scale 1:106670).

Field surveys were conducted at multiple forested sites within Cat Ba National Park and its surrounding areas during May and July 2025. Specimens were collected using three complementary methods: (i) direct hand collection without specialised tools; (ii) random sweeping of trees and shrubs along forest roads using an entomological sweep net; and (iii) beating of low branches and understory vegetation up to arm’s reach, with beetles dislodged onto a beating tray and subsequently collected using forceps. The geographic coordinates of all sampling sites are provided in Table 1 and illustrated in Fig 1.

**Table 1:** Sampling localities and specimen numbers on Cat Ba Island.

Locality	Latitude	Longitude	Elevation (m)	Number of specimens
1	20.794449	106.987841	40	6
2	20.78678	107.001459	41	9
3	20.782196	107.009909	41	11
4	20.779497	107.012511	40	5
5	20.787253	107.00663	35	12
6	20.794971	106.983926	30	1
7	20.783644	106.978182	28	9
8	20.788706	106.975729	24	11
9	20.778289	106.979291	25	10
10	20.775423	106.981662	26	6
11	20.800223	106.98364	29	10
12	20.808576	106.978387	32	6
13	20.809014	106.977458	33	7
14	20.830347	106.966967	32	2
15	20.833437	106.979561	30	3
16	20.793992	106.993825	48	5
17	20.793961	106.993768	58	1
18	20.794256	106.997154	89	1
19	20.79346	106.99881	166	1
20	20.799976	107.041338	11	1
21	20.803674	107.038466	15	1
**Total**				**118**

Maps were created using data from Natural Earth (public domain, http://www.naturalearthdata.com/) and visualized in QGIS

To minimise potential impacts on local populations and to comply with conservation guidelines, sampling at each site was restricted to a maximum of three individuals per morphospecies. All specimens collected from each locality were preserved separately in vials containing 90% ethanol and were labelled with the date of collection, locality, and collector’s name.

Field sampling was conducted in accordance with Vietnamese regulations. No specific permits were required for insect collection because the study did not involve protected or endangered species, and sampling was carried out in areas without restricted access. Permission to access sampling sites within Cat Ba National Park and surrounding areas was granted by local authorities.

### Morphological identification and integrative taxonomic framework

Specimens were initially sorted into morphospecies based on diagnostic external morphological characters. Species-level identifications were achieved for morphospecies where feasible through detailed comparison with published taxonomic keys, original descriptions, and authoritative taxonomic revisions [20–38].

Morphological species hypotheses were subsequently assessed using DNA barcoding data where such data were available. For morphospecies represented by successfully sequenced specimens, mitochondrial COI sequences were compared with Barcode Index Numbers (BINs) in the Barcode of Life Data System (BOLD) to evaluate concordance between morphological delimitation and molecular clustering. In contrast, for morphospecies lacking DNA barcode data—either due to unsuccessful amplification or the absence of reference sequences in BOLD—species delimitation relied exclusively on morphological evidence.

Accordingly, molecular data were used as an auxiliary line of evidence rather than a universal criterion for species identification. This combined but flexible framework allowed integrative taxonomic inference where possible, while maintaining a morphology-based approach for taxa without available genetic information.

Taxonomic nomenclature at the family and subfamily levels follows [39], whereas generic and species-level classifications are adopted from [40].

Representative specimens were photographed using a Nikon DS-Fi3 digital camera mounted on a Nikon SMZ800N stereomicroscope. Image stacks obtained from multiple focal planes were processed in NIS-Elements and combined using Helicon Focus 7 to generate fully focused composite images suitable for detailed morphological examination.

### DNA extraction, amplification and sequencing

Genomic DNA was extracted from whole specimens using the QIAamp® DNA Investigator Kit (QIAGEN) in accordance with the manufacturer’s instructions. Molecular analyses focused on the mitochondrial cytochrome c oxidase subunit I (COI) gene.

Mitochondrial COI was selected as the primary genetic marker because it represents the most widely used standard for insect DNA barcoding and allows direct comparability with existing reference data in BOLD and GenBank. Although reliance on a single mitochondrial locus may limit phylogenetic resolution, the present study employs COI as a pragmatic and widely accepted marker for species delimitation, baseline genetic characterisation and exploratory evolutionary analyses in a data-deficient system.

An approximately 658 bp fragment of COI was amplified using the universal primer pair LCO1490 and HCO2198 [41]. Polymerase chain reactions (PCRs) were conducted in a total volume of 25 µl, containing 2.5 µl of 10 × reaction buffer (Evrogen, Russia), 0.5 µl of 10 mM dNTP mix, 0.5 µl each of forward and reverse primers (10 µM), 1 µl of 25 mM Mg² ⁺ , 2 µl of template DNA, 0.2 µl of thermostable Taq DNA polymerase (Evrogen, Russia), and nuclease-free deionised water to volume.

Thermal cycling conditions comprised an initial denaturation step at 94 °C for 3 min, followed by 35 cycles of denaturation at 94 °C for 30 s, annealing at 42 °C for 40 s, and extension at 72 °C for 60 s, with a final extension at 72 °C for 5 min. Amplification success was assessed by electrophoresis on 1.5% agarose gels. Successfully amplified products were sequenced bidirectionally using the BigDye Terminator v3.1 Cycle Sequencing Kit (Applied Biosystems, Foster City, CA, USA) with the same primers employed for PCR.

Following DNA extraction, voucher specimens were air-dried, mounted, and labelled with unique voucher numbers. All voucher material was deposited in the Institute of Biology (IOB), Vietnam Academy of Science and Technology (VAST), to ensure traceability and long-term preservation.

Forward and reverse Sanger sequence reads were assembled, edited, and aligned using Geneious Prime 2025.2 (Biomatters Ltd.). The resulting sequences were deposited in GenBank (NCBI) under accession numbers PZ276027–PZ276050, PZ270350, PZ270351, PZ275966, PZ275967, PZ275968, PX170664, PX170665, PX170666, PX170669, and PX170674 and in the Barcode of Life Data System (BOLD v4) [42]. Automatic assignment of sequences to Barcode Index Numbers (BINs) was performed through the BOLD platform [43]. All sequence data are publicly available.

### Divergence time estimation

Twenty-five species of Chrysomelidae collected from Cat Ba Island were selected for divergence-time analyses based on the availability of high-quality, sufficiently long DNA sequences. Phylogenetic relationships and divergence times were inferred within a Bayesian framework using BEAST v10.5 [44]. Sequence evolution was modelled under the GTR + I nucleotide substitution model, with all parameters estimated from the data. Rate heterogeneity among lineages was accommodated using an uncorrelated lognormal relaxed molecular clock (UCLD), which allows substitution rates to vary independently across branches. Lineage diversification was modelled using a Birth–Death process.

Because no reliable fossil calibrations are currently available for the focal taxa, divergence-time estimation relied exclusively on molecular rate calibration. A prior on the mitochondrial substitution rate was applied to the COI gene, with a mean rate of 0.0115 substitutions per site per million years per lineage. This rate has been widely employed in beetle and insect phylogenetic studies and is commonly used for estimating interspecific divergence times [45,46]. Importantly, this value was implemented as a prior distribution on the clock model rather than as a fixed rate, thereby explicitly incorporating uncertainty associated with molecular rate variation.

Markov chain Monte Carlo (MCMC) analyses were run for a sufficient number of generations to ensure adequate sampling of the posterior distribution, with parameters sampled at regular intervals. Convergence diagnostics and mixing performance were examined in Tracer v1.7.2 [47], and all key parameters exhibited effective sample size (ESS) values > 200. Posterior distributions of trees were summarised as maximum clade credibility (MCC) trees after discarding the initial 25% of samples as burn-in using TreeAnnotator v10.5.0 [44].

The resulting time-calibrated phylogeny forms the basis for the divergence-time estimates presented in the Results section, with node ages and their associated 95% highest posterior density (HPD) intervals visualised and interpreted in an evolutionary and biogeographical context using FigTree v1.4.4 [48].

These analyses were conducted to place local species diversity within a broad temporal framework, rather than to test detailed macroevolutionary hypotheses or provide precise estimates of speciation and extinction dynamics. Given the moderate taxon sampling and reliance on a single mitochondrial marker, the results should be interpreted cautiously and are not intended as definitive tests of alternative macroevolutionary scenarios.

### Inference of Diversification Dynamics of Chrysomelidae on Cat Ba Island

Diversification dynamics of Chrysomelidae on Cat Ba Island were investigated using complementary maximum-likelihood (ML) and Bayesian approaches to ensure robustness of inference. All analyses were based on a time-calibrated (ultrametric) phylogenetic tree comprising 25 extant taxa, inferred from Bayesian divergence-time analyses. The tree was imported into R v. 4.4.2 [49] and verified to be ultrametric prior to diversification analyses. Branch lengths represent divergence times (in millions of years) and were assumed to adequately reflect the evolutionary history of the focal clade.

### Maximum-likelihood diversification analyses

ML analyses were conducted in R v. 4.4.2 using the package diversitree [50], which implements likelihood-based methods for estimating diversification parameters from time-calibrated phylogenies. Analyses were performed on the ultrametric tree, with likelihoods conditioned on survival of the clade to the present.

Two constant-rate diversification models were fitted. First, a Yule (pure-birth) model was applied, assuming a constant speciation rate (λ) through time and no extinction (μ = 0). Second, a constant-rate birth–death (BD) model was fitted, allowing both speciation (λ) and extinction (μ) to occur at constant rates. The Yule model included one free parameter (λ), whereas the BD model included two free parameters (λ and μ).

Model parameters were estimated by maximising the likelihood function using numerical optimisation. To ensure stable parameter estimation and avoid convergence on local optima, each model was fitted using multiple starting values for λ (and μ in the BD model). All optimisation runs converged on the same maximum-likelihood estimates.

Model fit was assessed using the Akaike Information Criterion (AIC), calculated as

AIC=2k−2logL, where k is the number of free parameters and log L is the maximum log-likelihood. Relative support for competing models was evaluated using differences in AIC (ΔAIC), with lower AIC values indicating better-supported models.

### Bayesian diversification analyses

To further explore diversification dynamics and to test for potential temporal heterogeneity in diversification rates, a Bayesian framework was implemented using the R v. 4.4.2 package TESS v. 3.0 [51]. Two alternative models were analysed: (1) a constant-rate birth–death model, assuming time-homogeneous speciation (λ) and extinction (μ) rates, and (2) a rate-shift birth–death model, allowing diversification rates to vary through time.

Analyses were conducted on the reconstructed phylogeny while explicitly accounting for incomplete taxon sampling via a sampling probability parameter. Posterior distributions of diversification parameters were estimated using Markov chain Monte Carlo (MCMC) sampling. Model fit was evaluated using posterior log-likelihoods and AIC values, with ΔAIC > 10 interpreted as strong support for the model with the lower AIC.

These analyses are intended to provide a coarse-grained characterisation of diversification patterns rather than precise estimates of speciation and extinction rates.

### Ancestral area reconstruction

Historical biogeographic analyses were conducted to infer ancestral range evolution and dispersal processes in Chrysomelidae using the R v. 4.4.2 package BioGeoBEARS [52]. Species distributions were coded into four discrete geographic areas: (1) Cat Ba Island, (2) mainland northern Vietnam, (3) adjacent regions of southern China, and (4) India. These areas were defined to reflect the major biogeographic units relevant to the evolutionary history of the studied lineages, capturing both continental source regions and insular systems while maintaining a tractable number of areas for likelihood-based inference.

Analyses were based on a mitochondrial COI dataset comprising 658 base pairs. A total of 75 Chrysomelidae species were included, with representatives from Cat Ba Island (25 species), mainland Vietnam (17 species), India (12 species), and mainland China (21 species). COI sequences for taxa from China, India, and nine species from mainland Vietnam were obtained from GenBank, whereas the remaining sequences were newly generated in this study (Table 2).

**Table 2:** COI sequences of Chrysomelidae used in this study.

No.	GenBank accession	Species	Country	Reference
1	MN346010	*Physosmaragdina nigrifrons*	China	GenBank
2	MN343857	*Basilepta leechi*	China	GenBank
3	MN433130	*Lilioceris cheni*	China	[53]
4	MN433209	*Lilioceris egena*	China	[53]
5	MH477480	*Altica fragariae*	China	[54]
6	KU697486	*Nisotra* sp.	China	[55]
7	KU697500	*Xuthea yunnanensis*	China	[55]
8	KU697479	*Longitarsus exsoletus*	China	[55]
9	MN344265	*Morphosphaera japonica*	China	GenBank
10	KC255428	*Plagiodera versicolora*	China	GenBank
11	KU697470	*Hemipyxis tonkinensis*	China	[55]
12	KU697469	*Hemipyxis plagioderoides*	China	[55]
13	KU697489	*Phyllotreta striolata*	China	[55]
14	MN344191	*Hemipyxis* sp.	China	GenBank
15	MN344779	*Liroetis postmaculatus*	China	GenBank
16	KC255446	*Nonarthra pulchrum*	China	[55]
17	KC255419	*Gonioctena olivacea*	China	[55]
18	MN345721	*Linaeidea placida*	China	GenBank
19	KC255440	*Sphenoraia bicolor*	China	[55]
20	MN345819	*Ophrida spectabilis*	China	GenBank
21	MF133458	*Agonita chinensis*	China	GenBank
22	MF140508	*Psylliodes* sp.	India	GenBank
23	MH198026	*Paridea* sp.	India	GenBank
24	MF495676	*Oides affinis*	India	GenBank
25	KT175603	*Phyllotreta striolata*	India	[56]
26	MF140486	*Arthrotus flavocincta*	India	GenBank
27	MN658891	*Callosobruchus maculatus*	India	GenBank
28	KJ195318	*Aspidimorpha sanctaecrucis*	India	GenBank
29	KX778635	*Hispa atra*	India	GenBank
30	KY769284	*Aspidimorpha furcata*	India	GenBank
31	KJ195316	*Chiridopsis cf. bipunctata*	India	GenBank
32	KJ195312	*Chiridopsis undecimnotata*	India	GenBank
33	KJ195313	*Laccoptera foveolata*	India	GenBank
34	PZ275964	*Demotina* sp.2	Vietnam	This study
35	PZ275963	*Demotina* sp.3	Vietnam	This study
36	PZ275965	*Demotina* sp.4	Vietnam	This study
37	PZ276055	*Menippus* sp.	Vietnam	This study
38	PZ276054	*Menippus* sp.	Vietnam	This study
39	PZ276053	*Menippus* sp.	Vietnam	This study
40	PZ276052	*Pyrrhalta* sp.	Vietnam	This study
41	MN845117	*Lema praeusta*	Vietnam	[57]
42	MN845116	*Lema saigonensis*	Vietnam	[57]
43	MN845114	*Lema demangei*	Vietnam	[57]
44	MN845119	*Aulacophora mouhoti*	Vietnam	[57]
45	MN845118	*Aulacophora indica*	Vietnam	[57]
46	MN845120	*Aspidimorpha dorsata*	Vietnam	[57]
47	MN845123	*Aspidimorpha furcata*	Vietnam	[57]
48	MN845124	*Chiridopsis bowringii*	Vietnam	[57]
49	MN845125	*Cassida circumdata*	Vietnam	[57]

Ancestral range estimation was performed using the likelihood-based Dispersal–Extinction–Cladogenesis (DEC) model and its extension incorporating founder-event speciation (DEC + J**).** Both models were implemented on a time-calibrated ultrametric maximum clade credibility (MCC) tree inferred in BEAST, with branch lengths proportional to divergence time. Species distributions were coded as presence–absence across the predefined areas, and the maximum range size was constrained to three areas per lineage to reflect biologically realistic distributions.

All analyses assumed a fixed dispersal matrix with constant dispersal and extinction rates through time. Model parameters were estimated using maximum likelihood, and model fit was evaluated by comparing log-likelihood values and the Akaike Information Criterion corrected for small sample size (AICc). The comparison between DEC and DEC + J was explicitly designed to assess whether allowing cladogenetic founder-event dispersal improved model fit and altered inferred ancestral ranges, particularly in the context of continental–island biogeographic systems.

Rather than conducting an exhaustive comparison across all available BioGeoBEARS models, our analytical framework focused on evaluating the relative support for gradual range evolution (DEC) versus scenarios that additionally permit jump dispersal at speciation (DEC + J). This targeted approach was chosen to directly test hypotheses regarding the role of founder-event speciation in shaping present-day distribution patterns, while acknowledging ongoing discussions regarding the interpretation of the J parameter. Accordingly, results from DEC + J are interpreted cautiously and used primarily to identify qualitative spatial patterns rather than to infer explicit dispersal mechanisms.

The inclusion of the DEC + J model is intended to improve descriptive fit to the observed distribution patterns rather than to imply explicit founder-event dispersal processes. In line with recent discussions on the interpretation of the J parameter, results from DEC + J are treated as phenomenological and are used primarily to highlight qualitative spatial structuring of lineages rather than to infer specific dispersal mechanisms.

## Results

### Species diversity and taxonomic composition of Chrysomelidae on Cat Ba Island

**Fig 2:**
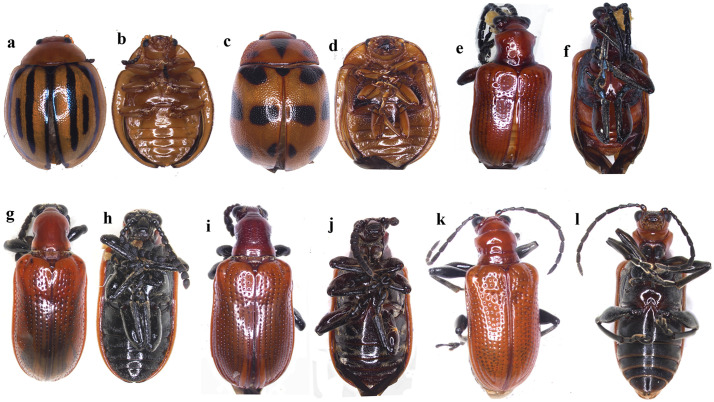
Dorsal and ventral habitus of leaf beetle species. (a, b) *P. septemvittata*; (c, d) *G. tredecimaculata*; (e, f) *L. cyaneicollis*; (g, h) *L. egena*; (i, j) *L. impressa*; (k, l) *L. (L.) rondoniana*.

**Fig 3:**
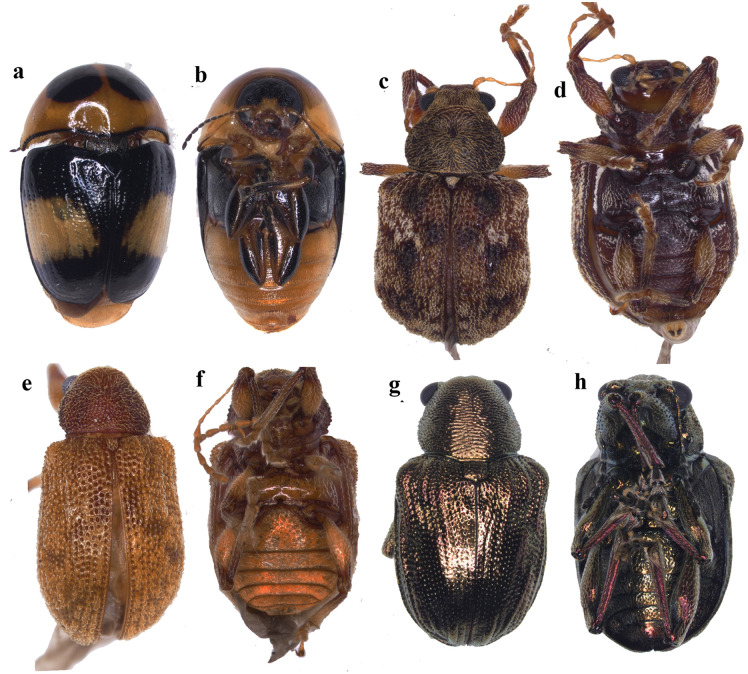
Dorsal and ventral habitus of leaf beetle species. (a, b) *Melixanthus* sp.; (c, d) *Demotina* sp. 1; (e, f) *Pseudometaxis* sp.; (g, h) *H. granulosa*.

**Fig 4:**
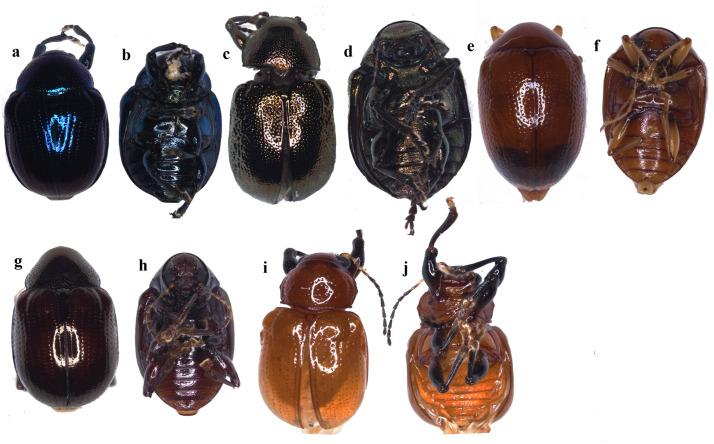
Dorsal and ventral habitus of leaf beetle species. (a, b) *Colaspoides* sp.; (c, d) *Colasposoma* sp.; (e, f) *Basilepta* sp.; (g, h) *Cleoporus* sp.; (i, j) *Pagria* sp.

**Fig 5:**
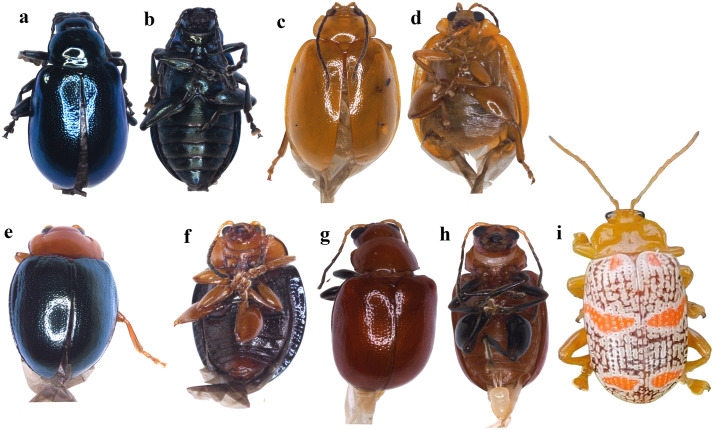
Dorsal and ventral habitus of leaf beetle species. (a, b) *Altica* sp.; (c, d) *Hemipyxis* sp.; (e, f) *N. chrysomeloides*; (g, h) *Paradibolia* sp.; (i) *P. dalmani*.

**Fig 6:**
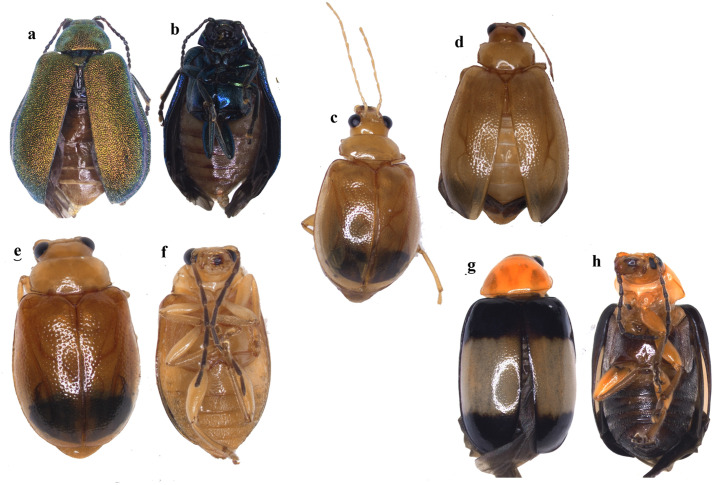
Dorsal and ventral habitus of leaf beetle species. (a, b) *Clitenella* sp.; (c) *Dercetina* sp. 1; (d) *Dercetina* sp. 2; (e, f) *Dercetina* sp. 3; (g, h) *Morphosphaera* sp.

**Fig 7:**
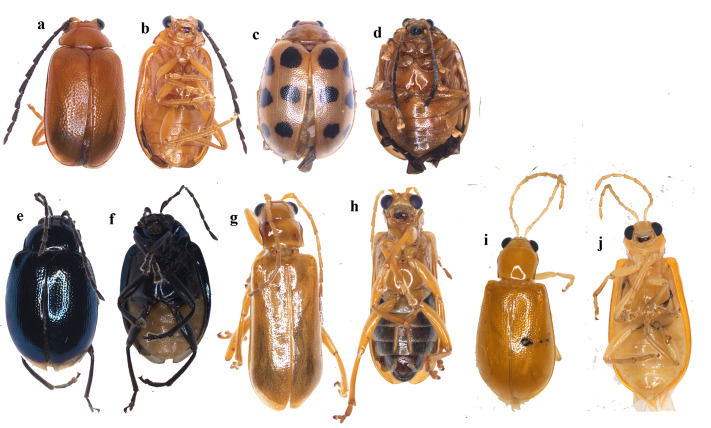
Dorsal and ventral habitus of leaf beetle species. (a, b) *Sermyloides* sp.; (c, d) *S. nebulosa*; (e, f) *Cassena* sp.; (g, h) *H. unicolor*; (i, j) *K. unicolor*.

**Fig 8:**
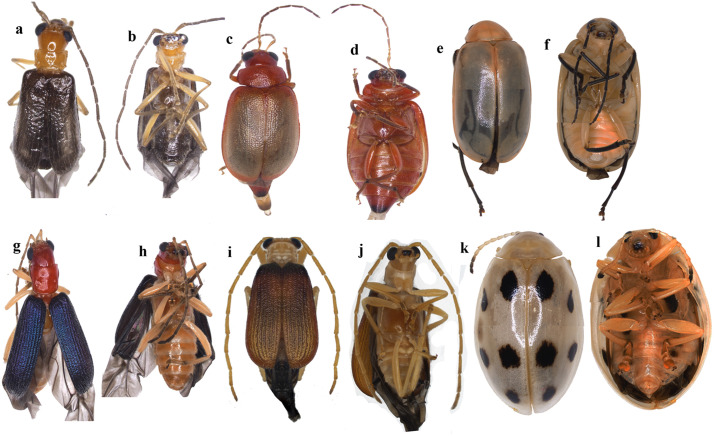
Dorsal and ventral habitus of leaf beetle species. (a, b) *Mimastra* sp.; (c, d) *Monolepta* sp. 1; (e, f) *Monolepta* sp. 2; (g, h) *T. (T.) bowringii*; (i, j) *T. (P.) nigribasalis*; (k, l) *O. decempunctata*.

All specimens collected during field surveys were assigned to morphospecies operational taxonomic units (OTUs). In total, 36 morphospecies OTUs were recognised, of which 16 were identified to species level, while the remaining OTUs were identified to genus level. These OTUs represent 30 genera belonging to five subfamilies of Chrysomelidae recorded from Cat Ba Island (Table 3, Figs 2–8).

**Table 3:** List of Chrysomelidae species, BINs, and specimen information collected in Cat Ba island.

No.	Taxon	BIN (BOLD)	Figure	BOLD status	No. of specimens	Locality
	Subfamily CHRYSOMELINAE Latreille, 1802					
	Tribe *Chrysomelini* Latreille, 1802					
	Subtribe Chrysomelina Latreille, 1802					
	Genus *Plagiodera Chevrolat in Dejean, 1836*					
1	*Plagiodera septemvittata* (Fabricius, 1798)	BOLD:AHF5762	Fig 2a–b	New BIN	2	19
	Subtribe *Gonioctenina* Motschulsky, 1860					
	Genus *Gonioctena* Chevrolat in Dejean, 1836					
2	*Gonioctena tredecimaculata* (Jacoby, 1888)	BOLD:AHF5763	Fig 2c–d	New BIN	1	1
	Subfamily CRIOCERINAE					
	Tribe *Criocerini* Latreille, 1804					
	Genus *Lilioceris* Reitter, 1912					
3	*Lilioceris cyaneicollis* Pic, 1916	BOLD:AHH2351	Fig 2e–f	New BIN	3	9
4	*Lilioceris egena* Weise, 1922	BOLD:AHF4619	Fig 2g–h	New BIN	3	10
5	*Lilioceris impressa* (Fabricius, 1787)	BOLD:AHH2350	Fig 2i–j	New BIN	3	11
	Tribe *Lemiini* Gyllenhal, 1813					
	Genus *Lema* Fabricius, 1798					
6	*Lema (Lema) rondoniana* Kimoto & Gressitt, 1979	BOLD:ACZ3991	Fig 2k–l	Existing BIN	2	4
	Subfamily CRYPTOCEPHALINAE					
	Tribe *Cryptocephalini* Gyllenhal, 1813					
	Subtribe *Cryptocephalina* Gyllenhal, 1813					
	Genus *Melixanthus* Suffrian, 1854					
7	*Melixanthus* sp.	–	Fig 3a–b	–	1	12
	Subfamily EUMOLPHINAE					
	Tribe *Bromiini* Baly, 1865					
	Genus *Demotina* Baly, 1863					
8	*Demotina* sp. 1	BOLD:AHF2298	Fig 3c–d	New BIN	1	13
	Genus *Pseudometaxis* Jacoby, 1900					
9	*Pseudometaxis* sp.	–	Fig 3e–f	–	1	14
	Genus *Heteraspis* Chevrolat in Dejean, 1836					
10	*Heteraspis granulosa* (Baly, 1867)	BOLD:ADE7488	Fig 3g–h	Existing BIN	2	10
	Tribe *Eumolpini* Hope, 1840					
	Genus *Colaspoides* Laporte, 1833					
11	*Colaspoides* sp.	BOLD:AFD5872	Fig 4a–b	Existing BIN	2	9, 11
	Tribe *Euryopini* Chapuis, 1874					
	Genus *Colasposoma* Laporte, 1833					
12	*Colasposoma* sp.	BOLD:AFQ0395	Fig 4c–d	Existing BIN	2	9, 11
	Tribe *Typophorini* Baly, 1865					
	Genus *Basilepta* Baly, 1860					
13	*Basilepta* sp.	BOLD:AHF2299	Fig 4e–f	New BIN	2	11
	Genus *Cleoporus* Lefèvre, 1884					
14	*Cleoporus* sp.	BOLD:AHF2297	Fig 4g–h	New BIN	3	11
	Genus *Pagria* Lefèvre, 1884					
15	*Pagria* sp.	BOLD:AHF2300	Fig 4i–j	New BIN	3	16
	Subfamily *GALERUCINAE*					
	Tribe *Alticini* Newman, 1834					
	Genus *Altica* Geoffroy, 1762					
16	*Altica* sp.	BOLD:AHF9227	Fig 5a–b	New BIN	3	12
	Genus *Hemipyxis* Dejean, 1836					
17	*Hemipyxis* sp.	BOLD:AHF9228	Fig 5c–d	New BIN	8	3, 4, 5
	Genus *Nisotra* Baly, 1864					
18	*Nisotra chrysomeloides* Jacoby, 1885	BOLD:ADH7382	Fig 5e–f	Existing BIN	3	2
	Genus *Paradibolia* Baly, 1875					
19	*Paradibolia* sp.	BOLD:AHF9226	Fig 5g–h	New BIN	1	14
	Genus *Podontia* Dalman, 1824					
20	*Podontia dalmani* Baly, 1865	BOLD:AGX2433	Fig 5i	New BIN	1	3
	Tribe *Galerucini* Latreille, 1802					
	Genus *Clitenella* Laboissière, 1927					
21	*Clitenella* sp.	BOLD:AHF2039	Fig 6a–b	New BIN	2	13
	Tribe *Hylaspini* Chapuis, 1875					
	Genus *Dercetina* Gressitt & Kimoto, 1963					
22	*Dercetina* sp. 1	BOLD:AHF2040	Fig 6c	New BIN	2	9
23	*Dercetina* sp. 2	BOLD:AHF2036	Fig 6d	New BIN	2	7
24	*Dercetina* sp. 3	BOLD:AHF9051	Fig 6e–f	–	1	18
	Genus *Morphosphaera* Baly, 1861					
25	*Morphosphaera* sp.	–	Fig 6g–h	–	2	7
	Genus *Sermyloides* Jacoby, 1884					
26	*Sermyloides* sp.	BOLD:AGV6347	Fig 7a–b	New BIN	2	5
	Genus *Sphenoraia* Clark, 1865					
27	*Sphenoraia nebulosa* (Gyllenhaal, 1808)	BOLD:AHF2035	Fig 7c–d	New BIN	15	1, 2, 3, 5, 6,7, 8, 16
	Tribe *Luperini* Gistel, 1848					
	Subtribe *Luperina* Gistel, 1848					
	Genus *Cassena* Weise, 1892					
28	*Cassena* sp.	BOLD:AGW9679	Fig 7e–f	New BIN	1	5
	Genus *Hoplasoma* Jacoby, 1884					
29	*Hoplasoma unicolor* (Illiger, 1800)	BOLD:AHG1200	Fig 7g–h	New BIN	14	2, 3, 8, 9, 10,11,12, 20, 21
	Genus *Kanarella* Jacoby, 1896					
30	*Kanarella unicolor* Jacoby, 1896	–	Fig 7i–j	–	12	2, 3, 5, 7, 8
	Genus *Mimastra* Baly, 1865					
31	*Mimastra* sp.	–	Fig 8a–b	–	2	9
	Genus *Monolepta* Chevrolat in Dejean, 1836					
32	*Monolepta* sp. 1	BOLD:AHF2041	Fig 8c–d	New BIN	2	13
33	*Monolepta* sp. 2	BOLD:AFN6221	Fig 8e–f	Existing BIN	2	12
	Genus *Trichobalya* Weise, 1924					
34	*Trichobalya bowringii* Baly, 1890	BOLD:AGV0546	Fig 8g–h	New BIN	3	15
35	*Trichobalya (Paratrichobalya) nigribasalis* Nguyen, 2025	BOLD:AGV0544	Fig 8i–j	New BIN	2	13
	Tribe *Oidini* Laboissière, 1921					
	Genus *Oides* Weber, 1801					
36	*Oides decempunctata* (Billberg, 1808)	BOLD:AGV6346	Fig 8k–l	New BIN	6	16, 17

Species richness differed markedly among subfamilies. Galerucinae was the most diverse subfamily, comprising 21 species distributed across 17 genera. Eumolpinae was the second most diverse group, with eight species in eight genera, followed by Criocerinae, with four species in two genera, and Chrysomelinae, with two species in two genera. Cryptocephalinae exhibited the lowest diversity, being represented by a single species in one genus.

Most morphospecies showed restricted spatial distributions across sampling sites. H. unicolor was the most widely distributed species, occurring at 9 of the 21 sampling sites, followed by S. nebulosa (8/21 sites) and K. unicolor (5/21 sites). Hemipyxis sp. was recorded from three sampling sites, whereas Colaspoides sp., Colasposoma sp., and O. decempunctata were each collected from two sites. The remaining 29 morphospecies OTUs were each recorded from a single sampling locality.

### DNA barcoding and genetic diversity

All OTUs were subjected to DNA extraction, amplification, and sequencing of the 658-bp fragment of the mitochondrial cytochrome c oxidase subunit I (COI) gene. However, successful amplification and sequencing were achieved for only 31 OTUs.

These sequences were submitted to the Barcode of Life Data System (BOLD) and were assigned to 31 Barcode Index Numbers (BINs). Among them, 25 were singletons and constitute newly recorded BINs in BOLD generated by this study (Table 3).

Analysis of sequence composition in BOLD showed that the mean nucleotide frequencies were 16.17% for G, 16.46% for C, 29.66% for A, and 35.71% for T. The mean GC content across sequences was 34.64%, with a marked GC bias at the first codon position (mean = 44.12%).

### Divergence time estimates

The relaxed clock model was strongly supported, with a high coefficient of variation (CV ≈ 0.55), rejecting a strict molecular clock. Likelihood values were stable across analyses, and nucleotide substitution parameters were highly consistent, indicating robust phylogenetic signal. Effective sample size (ESS) values for all key parameters exceeded 200, indicating adequate convergence of the Markov chain Monte Carlo analyses.

**Fig 9:**
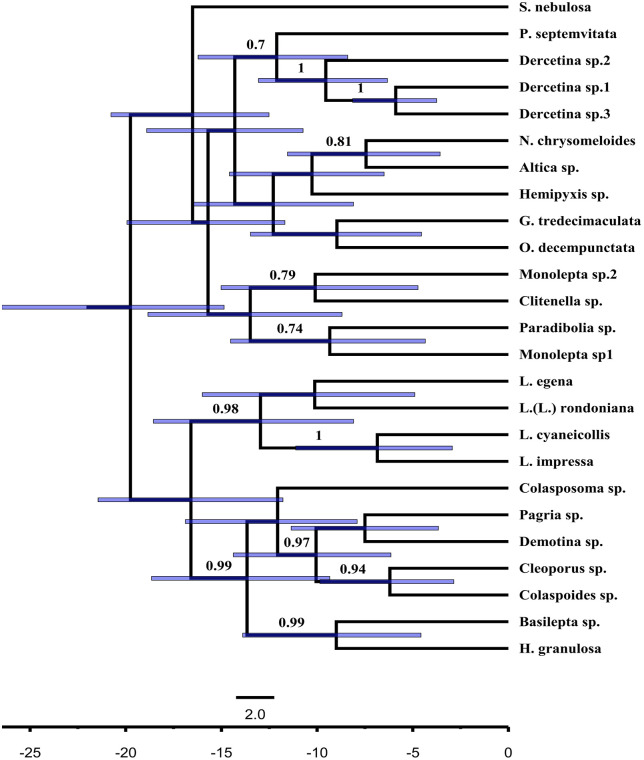
Maximum clade credibility (MCC) tree inferred using BEAST under a Birth–Death speciation model with an uncorrelated lognormal relaxed molecular clock. Node ages are shown as mean estimates. The estimated age of the root is 19.75 Ma (95% highest posterior density [HPD]: 14.86–26.47 Ma). Horizontal bars at nodes represent 95% HPD intervals of divergence times. Numbers at nodes indicate posterior probabilities. Node ages are colour-coded according to the scale shown on the left, and the time scale (Ma) is shown along the bottom axis.

Divergence time estimates obtained from BEAST analyses are summarised in the maximum clade credibility tree (Fig 9). The most recent common ancestor of the study group was dated to approximately 19.75 Ma (95% HPD: 14.86–26.47 Ma), corresponding to the early–middle Miocene. Major lineages diverged shortly after the origin of the group, followed by successive diversification toward the present. Deeper nodes exhibited broader uncertainty, whereas more recent splits showed narrower credibility intervals.

### Diversification dynamics

#### Maximum-likelihood constant-rate models.

ML analyses revealed broadly similar patterns of lineage accumulation under both constant-rate models. Under the Yule (pure-birth) model, the maximum log-likelihood was −26.54, with an estimated speciation rate of λ ≈ 0.079. The constant-rate birth–death model yielded a slightly lower maximum log-likelihood (−26.78), with an estimated speciation rate of λ ≈ 0.086 and an extinction rate μ close to zero.

Model comparison based on AIC slightly favoured the Yule model (ΔAIC = 2.48), providing moderate support for the simpler pure-birth model (Table 4).

**Table 4:** Comparison of diversification models under maximum likelihood and Bayesian frameworks. Diversification parameters were estimated using both maximum likelihood (ML) and Bayesian approaches. ML analyses compared Yule (pure-birth) and constant-rate birth–death models, whereas Bayesian analyses were conducted using TESS under constant-rate and rate-shift birth–death models. Reported parameters include speciation rate (λ), extinction rate (μ), and net diversification rate (r = λ − μ). Values in parentheses indicate 95% highest posterior density (HPD) intervals for Bayesian estimates. Akaike Information Criterion (AIC) and ΔAIC values were calculated within each analytical framework. Log-likelihood values are not directly comparable between ML and Bayesian approaches due to differences in model conditioning.

Framework	Model	Log-likelihood	AIC	ΔAIC	Speciation rate (λ)	Extinction rate (μ)	Net diversification rate (r)	Notes
**Maximum likelihood**	Yule (pure-birth)	−26.54	55.08	0.00	0.079	0 (fixed)	0.079	Best-supported ML model; no extinction signal
**Maximum likelihood**	Constant-rate birth–death	−26.61	57.23	2.15	0.086	6 × 10 ⁻ ⁷	≈0.086 (μ ≈ 0)	Extinction not supported
**Bayesian (TESS)**	Constant-rate birth–death	−81.02	166.41	0.00	0.104 (0.0645–0.1400)	0.0177 (0.00022–0.0502)	0.086 (0.0453–0.1282)	Better supported than rate-shift model
**Bayesian (TESS)**	Rate-shift birth–death	−82.97	171.96	5.55	0.130 (0.008–0.318)	0.050 (0.00001–0.125)	0.080 (−0.090–0.300)	Broad HPD including negative values; parameters poorly constrained

#### Bayesian analyses using TESS.

Bayesian analyses implemented in TESS further supported a constant-rate birth–death model over a rate-shift model. The constant-rate model achieved a higher posterior log-likelihood and a substantially lower AIC, whereas the rate-shift model was associated with broader HPD intervals and increased parameter uncertainty.

Under the constant-rate model, the posterior mean speciation rate was λ = 0.104 (95% HPD: 0.0645–0.140), while extinction rates were comparatively low (μ = 0.0177; 95% HPD: 0.00022–0.0502), yielding a net diversification rate of 0.086 (95% HPD: 0.0453–0.1282). In contrast, the rate-shift model showed broad HPD intervals for all parameters, including negative values of net diversification (r), indicating substantial uncertainty and poor parameter identifiability. (Table 4).

Both analytical frameworks (ML and Bayesian analyses using TESS) broadly support the conclusion that diversification in the study lineage is primarily driven by speciation, with extinction being weakly supported and poorly constrained.

#### Inferred biogeographic history of Chrysomelidae on Cat Ba Island.

Model comparison based on likelihood and information criteria favoured the DEC + J model over the DEC model (Table 5). The DEC + J model achieved a higher log-likelihood and a lower AICc value despite including one additional free parameter.

**Table 5:** Comparison of biogeographic models implemented in BioGeoBEARS. Models were evaluated using log-likelihood (LnL), number of free parameters (k), Akaike Information Criterion (AIC), and corrected AIC (AICc). The DEC + J model shows substantially better fit to the data than the DEC model.

Model	Log-likelihood (LnL)	k	AIC	AICc
DEC + J	−95.38	3	196.75	190.75
DEC	−140.92	2	285.83	281.83

Under both models, ancestral range reconstructions inferred predominantly single-area ancestral ranges, with Cat Ba Island and adjacent mainland regions frequently reconstructed at deep and intermediate nodes. The DEC + J model inferred more geographically restricted ancestral ranges at several internal nodes compared with the DEC model. Taken together, the inferred patterns are consistent with spatially structured range inheritance and repeated dispersal into island regions, as captured by the DEC + J framework.

**Fig 10:**
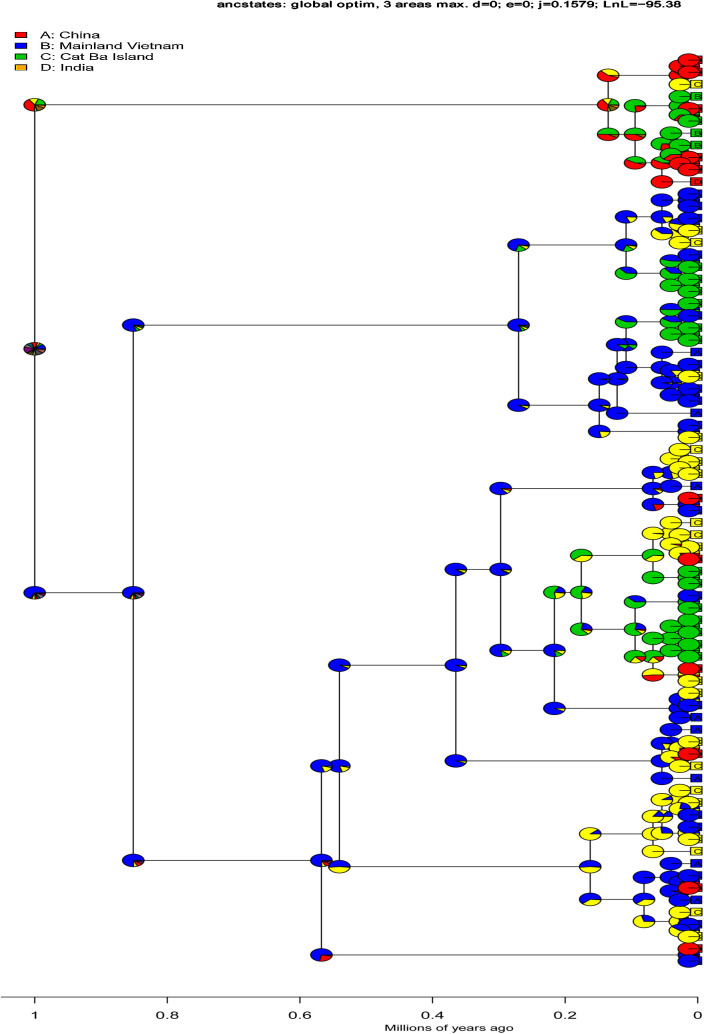
Ancestral area reconstruction inferred under the DEC + J model in BioGeoBEARS. Colors represent geographic areas: A = China, B = mainland Vietnam, C = Cat Ba island, and D = India. Pie charts at nodes indicate relative probabilities of ancestral ranges. The reconstruction highlights the predominance of Cat Ba Island and China as ancestral areas and supports patterns consistent with founder-event-like processes.

Ancestral state reconstructions under the DEC + J model are illustrated in the BioGeoBEARS plot (Fig 10). Cat Ba Island (A) and China (C) were most frequently inferred as ancestral areas at deeper nodes, whereas mainland Vietnam (B) was mainly reconstructed at shallow nodes. India (D) was inferred sporadically and did not represent a dominant ancestral region. Most nodes were reconstructed as single-area states rather than widespread multi-area distributions.

## Discussion

The present study provides an integrative assessment of species diversity, genetic structure, evolutionary history and biogeographic patterns of Chrysomelidae on Cat Ba Island, a continental shelf island in northern Vietnam. By combining morphological surveys, DNA barcoding, divergence-time estimation and model-based biogeographic analyses, the results offer a coherent framework for understanding how geological history, dispersal processes and ecological heterogeneity have shaped an insular leaf beetle assemblage.

The present study documents a diverse assemblage of Chrysomelidae on Cat Ba Island, comprising 36 morphospecies OTUs assigned to 30 genera and five subfamilies. This level of diversity is comparable to that reported from other island faunas in Vietnam, such as Phu Quoc Island with 44 OTUs [58], as well as islands in other tropical regions, including a west coast island of Sabah, Malaysia (68 OTUs [59]), and the island of St Vincent (47 species [60]).

Galerucinae was the most species-rich subfamily, accounting for more than half of the recorded OTUs. This dominance is consistent with both regional and global patterns, as Galerucinae is widely recognised as the most diverse and ecologically versatile lineage within Chrysomelidae, particularly in forested habitats [58,59,61]. The high generic diversity observed within Galerucinae on Cat Ba Island further reflects the broad host-plant associations and ecological plasticity characteristic of this group.

Eumolpinae represented the second most diverse subfamily, with eight species distributed across eight genera. Members of this subfamily are predominantly associated with woody vegetation and forest understorey plants, habitats that are well developed within the limestone and evergreen forests of Cat Ba Island. Comparable levels of Eumolpinae diversity have been reported from other tropical forest systems in the region [14]. In contrast, Chrysomelinae and Cryptocephalinae were represented by relatively few taxa. The low diversity of Cryptocephalinae, represented by a single species, may reflect both genuine ecological rarity and limitations of sampling, as many species are canopy-dwelling or exhibit seasonal activity patterns that reduce detectability [62,63].

At the spatial scale of the island, most morphospecies exhibited restricted distributions and were recorded from a single sampling locality. Such patterns are commonly observed in tropical beetle assemblages and are often attributed to habitat heterogeneity, patchy host-plant distribution and limited dispersal ability [64,65]. Nevertheless, several species showed relatively broad distributions across Cat Ba Island. H. unicolor, S. nebulosa and K. unicolor were recorded from multiple sampling sites, suggesting broader ecological tolerances and the ability to exploit widely distributed host plants. Similar contrasts between widespread and narrowly distributed species have been reported in leaf beetle communities from other Southeast Asian islands and mainland regions [58,59].

Species such as Hemipyxis sp., Colaspoides sp., Colasposoma sp. and O. decempunctata displayed intermediate distribution ranges, occurring at two or three sampling sites. These taxa may be more closely associated with specific habitat conditions or host plants, although further ecological data are required to clarify these relationships. Overall, the combination of a small number of widespread species and a large proportion of narrowly distributed taxa suggests that the chrysomelid fauna of Cat Ba Island is shaped by both dispersal ability and habitat specialisation, a pattern characteristic of insular ecosystems where geographic isolation and environmental heterogeneity promote high beta diversity [3,4].

To complement morphological diversity patterns, DNA barcoding of the COI gene generated sequence data for 31 species, resulting in the assignment of 31 Barcode Index Numbers (BINs), of which 25 were singleton BINs newly recorded in the Barcode of Life Data Systems (BOLD). The high proportion of singleton and novel BINs clearly reflects a pronounced deficiency of publicly available DNA barcode data for Vietnamese leaf beetles. Such gaps in reference databases constrain accurate species delimitation, hinder comparative biogeographic analyses, and reduce the effectiveness of biodiversity assessments in the region. From a conservation perspective, the incomplete documentation of Chrysomelidae diversity is particularly concerning for island ecosystems such as Cat Ba, where habitat fragmentation and anthropogenic pressures may disproportionately affect narrowly distributed or cryptic taxa. By contributing new barcode records to BOLD, this study represents an important step towards closing these data gaps and provides an initial genetic reference framework to support taxonomic research, biodiversity monitoring, and conservation planning for the Chrysomelidae fauna of Vietnam [42,66,67]. The observed nucleotide composition and GC bias at the first codon position are consistent with patterns commonly reported for insect mitochondrial genomes and COI barcode datasets [58,59] supporting the reliability of the generated barcode data and reinforcing the utility of DNA barcoding as a tool for species delimitation and future taxonomic research [67].

These analyses were conducted to place the observed diversity within a broad temporal and evolutionary context, rather than to test detailed macroevolutionary hypotheses. Within this exploratory framework, COI-based temporal analyses suggest that the major lineages of Chrysomelidae represented on Cat Ba Island may have originated during the early–middle Miocene. Divergence-time estimation under a Birth–Death model with an uncorrelated lognormal relaxed clock suggests rejection of a strict molecular clock, reflecting substantial among-lineage rate heterogeneity typical of insect mitochondrial datasets [46,68]. The inferred age of the most recent common ancestor at approximately 19.75 Ma is consistent with a period of major tectonic and climatic reorganisation in Southeast Asia, including intensified uplift and the establishment of monsoonal climates [9,69]. Although Cat Ba Island itself emerged much later, these results are consistent with the possibility that diversification was initiated in mainland prior to the formation of the present-day island landscape.

Exploratory analyses of diversification dynamics further suggest that the assembly of the Cat Ba chrysomelid fauna may not have involved pronounced bursts of diversification. Both maximum-likelihood and Bayesian approaches are broadly consistent with relatively constant diversification rates through time, with low inferred extinction. Under such conditions, pure-birth or constant-rate birth–death models provide parsimonious and statistically robust descriptions of lineage accumulation, particularly for phylogenies of moderate size [70,71]. The lack of strong support for rate-shift models may indicate that diversification did not proceed via episodic radiations linked to discrete environmental perturbations or key innovations [72,73]. Instead, the positive net diversification rate is consistent with sustained lineage accumulation without strong evidence for diversity-dependent slowdown.

This pattern is broadly consistent with the geological history of continental shelf islands such as Cat Ba, where cyclical sea-level fluctuations during the Quaternary repeatedly connected and fragmented island and mainland habitats [7,10]. These dynamics likely facilitated dispersal and recolonisation, whereas interglacial isolation may have promoted divergence without leading to extensive in situ radiation. These dynamics are expected to produce relatively homogeneous diversification trajectories over time rather than pronounced rate heterogeneity.

Biogeographic reconstructions provide preliminary insight into the spatial processes underlying this evolutionary history. Model comparison favoured the DEC + J framework in a statistical sense over the standard DEC model, indicating that spatially discrete range inheritance patterns captured by founder-event processes provide a better statistical fit to the data [74]. Nevertheless, as highlighted by earlier methodological assessments, the biological meaning of the j parameter requires careful interpretation, especially in cases where dispersal and extinction estimates lie near their boundary conditions [75]. In this context, DEC + J is best viewed as a phenomenological model describing patterns of range inheritance rather than a literal representation of dispersal mechanisms.

Ancestral area reconstructions suggest predominantly single-area ancestral ranges, with Cat Ba Island and China most frequently reconstructed at deep and intermediate nodes. Mainland Vietnam was mainly inferred at shallow nodes, suggesting more recent colonisation events, whereas India appeared sporadically and did not constitute a dominant ancestral region. These patterns are consistent with a spatially structured evolutionary history characterised by recurrent colonisation and limited range expansion, rather than widespread ancestral distributions. The recurrent inference of Cat Ba Island and China as ancestral areas suggests that these regions may have played central roles in the assembly of the fauna through multiple, independent colonisation events rather than a single directional dispersal pathway [4].

Taken together, the temporal framework, diversification analyses and biogeographic reconstructions are broadly consistent with a scenario in which the chrysomelid fauna of Cat Ba Island originated from mainland lineages during the Miocene and was subsequently assembled through repeated dispersal events linked to changes in continental shelf configuration. Diversification appears to have proceeded gradually at broadly constant rates, while founder-event-like range inheritance may have contributed to the spatial structuring of lineages [74]. In such systems, alternating phases of connection and fragmentation may promote lineage persistence and incremental diversification rather than rapid adaptive radiations [4,76].

For phytophagous beetles, the pronounced ecological heterogeneity of Cat Ba Island, including limestone-dominated forests and a diverse flora of potential host plants, may have provided additional opportunities for ecological differentiation at local scales. These conditions are consistent with a model of gradual diversification driven by the interaction between geological dynamics and fine-scale ecological variation, rather than by single pulses of ecological opportunity.

Despite its integrative approach, this study is subject to limitations. Moderate taxon sampling and the absence of fossil calibrations may constrain the detection of subtle shifts in diversification dynamics [70,71]. Broader geographic sampling across Cat Ba Island and adjacent mainland regions would improve inference within a dynamic shelf-island context [7,10]. In addition, reliance on mitochondrial COI data alone may be sensitive to lineage-specific rate variation, introgression, incomplete lineage sorting, and purifying selection, all of which may obscure true species boundaries and evolutionary relationships [46,66,77,78]. Furthermore, phylogenetic patterns inferred from mitochondrial data may differ from those based on nuclear markers, a phenomenon known as mitonuclear discordance [79]. Recent studies have also shown that closely related species may exhibit little or no mtDNA divergence despite being clearly distinct at nuclear loci [80], highlighting the need for caution when interpreting COI-based results. Future analyses incorporating nuclear markers are therefore expected to substantially strengthen phylogenetic inference. Finally, integrating ecological data, particularly host-plant associations, would allow more explicit tests of how ecological differentiation interacts with dispersal and isolation to shape biogeographic patterns [4,15].

## Conclusions

This study presents the first integrative synthesis of species diversity, genetic structure and evolutionary history of Chrysomelidae on Cat Ba Island. The available evidence suggests that the present-day fauna is composed of lineages that originated on the mainland during the Miocene and were subsequently assembled through repeated episodes of dispersal and isolation associated with continental shelf dynamics.

Diversification patterns inferred from mitochondrial COI data are broadly consistent with relatively constant rates through time, while spatially structured range inheritance appears to have contributed to present-day distribution patterns. Although these inferences are necessarily coarse-grained, they provide a useful evolutionary context for interpreting current biodiversity patterns.

Overall, the baseline taxonomic, genetic and biogeographic data generated in this study constitute a reference framework for future biodiversity monitoring, conservation assessments and comparative studies of insular insect faunas in Southeast Asia.

## Permission to reproduce material from other sources

All figures and tables are original. Fig 1 was created using public domain data from Natural Earth and QGIS software; therefore, no permission is required.
