# Correction: Leaf beetle diversity on a Southeast Asian continental island: Taxonomy, DNA barcoding, and preliminary evolutionary insights from Cat Ba Island, Vietnam

**DOI:** 10.1371/journal.pone.0353452

**Published:** 2026-07-07

**Authors:** Dinh Thi Nguyen, Loan Thi Ho

In Fig 1, the Hoang Sa (Paracel Islands) and Truong Sa (Spratly Islands) archipelagos were omitted in the map of Vietnam. Please see the correct Fig 1 here.

**Fig 1:**
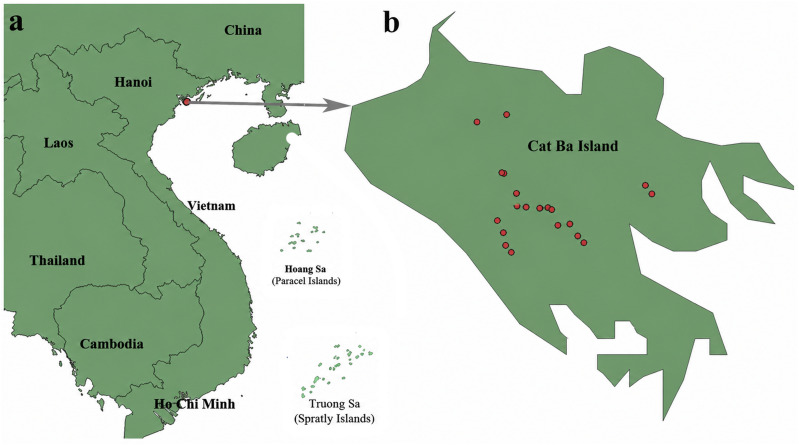
Sampling locations of Chrysomelidae on Cat Ba Island. **(a)** Regional map showing the location of Cat Ba Island within northern Vietnam (scale 1:9371119). **(b)** Detailed map of Cat Ba Island indicating sampling sites (scale 1:106670).

## References

[1] Nguyen DT, Ho LT. Leaf beetle diversity on a Southeast Asian continental island: Taxonomy, DNA barcoding, and preliminary evolutionary insights from Cat Ba Island, Vietnam. PLoS One. 2026;21(6):e0351706. doi: 10.1371/journal.pone.0351706 42313827 PMC13278452

